# The genetics of gene expression: comparison of linkage scans using two phenotype normalization methods

**DOI:** 10.1186/1753-6561-1-s1-s151

**Published:** 2007-12-18

**Authors:** Mariza de Andrade, Elizabeth J Atkinson, Brooke L Fridley, Ellen L Goode, Shannon McDonnell, Wen Liu-Mares, Kari G Rabe, Zhifu Sun, Susan L Slager

**Affiliations:** 1 Division of Biostatistics, Mayo Clinic College of Medicine, 200 First Street Southwest, Harwick 7, Rochester, Minnesota 55905, USA; 2 Division of Epidemiology, Mayo Clinic College of Medicine, 200 First Street Southwest, Harwick 7, Rochester, Minnesota 55905, USA

## Abstract

The goal of this paper is to investigate the effects of normalization procedures for expression data on linkage results. We selected the two most commonly used expression data extraction and normalization methods, Affymetrix global scaling and dChip invariant. After applying these two methods in 3554 expression phenotypes, we identified 45 phenotypes that were more likely to be genetic for either normalization procedure. A genome-wide linkage scan was performed on these expression values (45 phenotypes × 2 normalizations) using 2272 SNPs. Our results showed that: 1) the dChip normalization might inflate the LOD scores because the dChip normalization yielded LOD scores > 3.0 30% more frequently than the Affy normalization, and 2) the difference in LODs between the normalizations were not correlated with their heritabilities. In summary, we conclude, as have other published reports, that normalization methods play an important role in the linkage results, and that some significant linkage signals might be due to a specific normalization method.

## Introduction

There is great interest in understanding genetic factors related to variable expression of genes. Recently, several studies have shown the first evidence of heritability of mRNA between individuals [1-4]. By treating the expression phenotypes for each transcript (or probe) as a quantitative phenotype, a variance-components linkage analysis could be used [1-4]. The expectation is to detect linkage signals between the gene expression values and genomic regions. As pointed out by Chesler et al. [4], several issues plague these studies, including the choice of an appropriate normalization method and the selection of informative expression values. For the current analysis, we applied two data extraction and normalization methods, Affymetrix Microarray Suite with global scaling (Affy) and DNA chip analyzer's model-based expression indexes with invariant normalization (dChip), because they summarized probe-level signals using different approaches. The Affy method takes weighted average of a paired perfect and mismatch difference for a probe set, and the dChip method uses a model-based estimation at the probe level and accounts for probe-specific effects and outliers to derive a probe set expression. Thus, our primary goal was to investigate these two methods by studying their similarity in linkage signals, and our secondary goal was to investigate the similarity between our variance components-based linkage findings and those of Morley et al. [2], which were calculated using sib-pair regression based methodology and nuclear families only.

## Methods

### Data

The Genetic Analysis Workshop 15 (GAW15) Problem 1 Centre d'Etude du Polymorphisme Humain data consisted of 196 participants from 14 three-generation pedigrees with 14 individuals per family, 4 grandparents, 2 parents, and 8 offspring. Two hundred and seventy-six arrays, including data on 3554 probe sets on Affymetrix Human Focus Arrays, were provided by GAW15. These probe sets had been selected as those with greatest inter-individual variability from a total of 8500 probe sets [2].

### Feature-extraction and normalization methods

To assess the impact of data pre-processing on linkage analysis, we selected two feature-extraction and normalization methods, Affy and dChip. The data processed by the Affy method was provided by GAW15 and is the data set used by Morley et al. to identify significant linkage signals for genome-wide variation of gene expression [2]. These data were normalized by global scaling using Affymetrix Microarray Suite 5 with a target value of 500 [2]. The second feature extraction and normalization was conducted using dChip, a common approach found in microarray analytical packages that is described in detail by Li and Wong [5]. Briefly, we first normalized 276 arrays from each individual against an array with the median probe intensity using the invariant set algorithm. We then calculated the model-based expression indexes (MBEI) based on the perfect match probes (PM-only model) [5]. For both normalizations, the gene expression values for individuals with technical duplicates were averaged, and all expressions were log_2_-transformed.

### Selection of phenotype subsets

To increase the number of informative phenotypes, we excluded genes that had little variation in expression (standard deviation ≤ 0.3) and low call rates (absent calls > 90%) across samples; 3306 phenotypes (probe sets) remained. We further reduced the number by identifying those that were most likely to be genetic by calculating the heritability (*H*^2^) estimate (using the Splus/R library *multic *[6]) assuming a polygenic model for both the dChip and Affy normalizations. To reduce the number of phenotypes, we used a cutpoint of *H*^2 ^> 0.60 or when *H*^2 ^was significantly different from zero at α = 0.0001 for either normalization. This resulted in the inclusion of 45 phenotypes for linkage analysis.

### Genetic data

For a subset of subjects, including founders, we observed a large number of missing genotypes. Because of the increase of false positives due to tight linked markers [7], we reduced the extent of linkage disequilibrium between SNPs by removing SNPs with *r*^2 ^> 0.30 using ldSelect [8]. We then removed 2205 with Mendelian inconsistencies (0.5% of matings/genotypes). Thus, 2272 SNPs from a total of 2882 were used in the linkage analysis. Multipoint identity-by-descent (MIBD) sharing among pairs of relatives was calculated using the SIMWALK2 program [9].

### Quantitative trait linkage analysis

The 90 phenotypes (45 expression phenotype × 2 normalizations) were normally transformed using the van der Waerden rank transformation [10]. Variance-components linkage analyses were performed using the S-Plus library *multic *[6]. Sex was used as a covariate, consistent with Morley et al. [2]. We assessed evidence for linkage of the 90 phenotypes and considered "strong" linkage evidence for LOD score > 3.0, which assumes a genome-wide significance of 0.05.

## Results

The correlation of the coefficients of variation (CV) for 3554 phenotypes from the two data pre-processing methods was 0.64, indicating a reasonable agreement between the two procedures. In general, the Affy-normalized data had higher variation. There was high correlation between the two normalization methods (*r *= 0.97) for the 45 phenotypes selected for linkage analyses based on heritability (data not shown).

We compared the total number of LOD scores greater than 3.0 across the genome for each normalization method and found that: 1) the data from the dChip method led to twice as many positions with "strong" linkage evidence compared to the data from Affy, and 2) of the strong linkage findings, 49% were identified by both methods. The remaining findings between the two methods were discordant. For example, analysis of the activating transcription factor phenotype (205446_s_at) yielded 14 strong linkage signals (LOD > 3.0) across multiple chromosomes using the dChip method, but none were found using the Affy normalized method.

**Figure 1:**
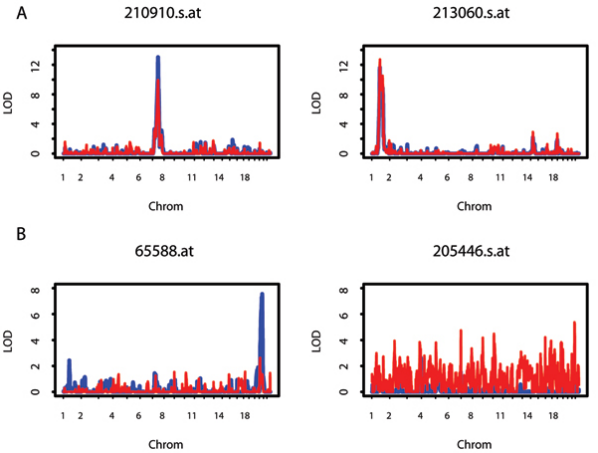
**Genome scans comparing the two normalization approaches**. A, two highly concordant expression phenotypes of the 25 expression phenotypes with LOD score differences < 3.0; B, two discordant expression phenotypes of the 20 expression phenotypes with LOD differences > 3.0.

We compared the LOD differences between the two data processing methods. We observed that 25 expression phenotypes had LOD score differences < 3.0 across the entire genome (Fig. 1A shows 2 of those 25), and 20 expression phenotypes had LOD differences > 3.0 in about 25% of the entire genome (Fig. 1B shows 2 of those 20). Three of these 20 (208151_s_at, 320_at, 65588_at) had most of the LOD difference > 3.0 on one chromosome (chromosomes 22, 6, 20, respectively), and 17 of the 20 had LOD difference > 3.0 distributed over multiple chromosomes. We observed no correlation between the difference in the LOD scores between the two normalization methods and their polygenic heritabilities (data not shown).

Table 1 shows the results between the two normalization methods we used and Morley's using a criterion of *p *< 10^-8 ^for significance with either normalization approach. Morley et al. used the Affy normalization method and their multipoint genome-wide linkage analysis was done using SIBPAL in S.A.G.E. [2] in which they used a sib-pair-based regression approach rather than variance-components methodology. Three of our seven strongest linkage signals matched those found by Morley et al.; four did not. For the remaining eight phenotypes identified by Morley et al., we were unable to compare the results because the specific expression phenotypes were not part of our final 45 selected phenotypes. Finally, we showed agreement in linkage signals between the Affy and dChip normalizations with seven expression phenotypes, and one disagreement (208151.x.at).

**Table 1:** Expression phenotypes with the strongest agreement and evidence of linkage for the two normalization procedures

				Affymetrix			dChip		
					
Gene^a^	Location^b^	Probe	Morley et al. *p*-value	LOD	*p*-value	Position (cM)	LOD	*p*-value	Position (cM)
*CHI3L2*	1p13.3	213060.s.at	10^-10^	11.6	<10^-12^	111.8	12.7	<10^-13^	111.9
*ZP3*	7q11.23	210910.at	<10^-9^	13	<10^-14^	75.6	9.9	<10^-11^	75.1
*PSPH*	7p15.2	205048.s.at	<10^-10^	6.1	<10^-7^	64.3	7.7	<10^-8^	64.3
									
*UGT2B17*	4q13	207245.at	---^c^	8.3	<10^-9^	62.3	9.6	<10^-10^	62.3
*LRAP*	5q15	219759.at	---	8.1	<10^-9^	99	7.6	<10^-8^	90.2
*HLA-DQB1*	6p21.3	209480.at	---	9	<10^-10^	36	12	<10^-13^	32.9
*HLA-DPB1*	6p21.3	201137.s.at	---	8.8	<10^-10^	31.6	8.7	<10^-9^	31.6
									
*DDX17*	22q13	208151.x.at	<10^-9^	5.9	<10^-7^	43.5	1.2	<10^-2^	43.2
*IL16*	15q26.3	209827.at	<10^-9^	---^c^	---	---	---	---	---
*ALG6*	1p31	219649.at	<10^-9^	---	---	---	---	---	---
*ICAP-1A*	2p25	-	<10^-11^	---	---	---	---	---	---
*HOMER1*	5q14.2	213793.at	<10^-10^	---	---	---	---	---	---
*HSD17B12*	11p11.2	217974.at	<10^-10^	---	---	---	---	---	---
*TM7SF3*	12q11-12	217869.at	<10^-11^	---	---	---	---	---	---
*TNFRSF11A*	18q22.1	207037.at	<10^-9^	---	---	---	---	---	---
*CBR1*	21q22.13	209213.at	<10^-10^	---	---	---	---	---	---
*DSCR2*	21q22.3	203405.at	<10^-10^	---	---	---	---	---	---

^a ^The first group of genes shows agreement between Morley et al. [2] and our findings. The second group represents our significant findings at the level of significance 10^-8 ^or less but not in Morley's. The third group represents significance in Morley's findings and not ours. The fourth group shows findings in Morley that we did not examine (*H*^2 ^< 0.6). The LOD represents the maximum LOD scores in the chromosome where the genes are located with its respective *p*-values and positions in cM.

^b ^Location was determined by the file HG-Focus_annot.csv.

^c ^--- indicates either the probes are not published by Morley et al. [2] or are not analyzed by us.

## Discussion

As reported recently by Chesler et al., normalization plays an important part in linkage analysis [4]. Our results indicate that the dChip normalization might inflate the LOD scores because here it yielded greater LOD scores than the Affy, and produced, on average, more evidence of linkage than the Affy despite the fact that the data from two normalizations were highly correlated. We also found that the difference in LOD scores between the normalizations was not correlated with their heritabilities. We also investigated the similarity between our linkage results and Morley et al. [2], and found agreement with three out of seven expression phenotypes that we considered.

## Conclusion

It remains unclear which pre-processing method for microarray data is the most appropriate to use for linkage analysis. The two procedures we assessed (Affy and dChip) were similar in most respects; however, in 16 of the 45 phenotypes, we observed a large difference between the two methods for at least one chromosome. Thus, further work is needed to examine the influence of data preparation and phenotype normalization methods on results of linkage analyses.

## Competing interests

The author(s) declare that they have no competing interests.
